# Prostate cancer risk reduction: promising prevention practices and insights

**DOI:** 10.1097/MS9.0000000000003451

**Published:** 2025-05-30

**Authors:** Emmanuel Ifeanyi Obeagu

**Affiliations:** Department of Biomedical and Laboratory Science, Africa University, Mutare, Zimbabwe

**Keywords:** lifestyle modifications, nutritional strategies, prevention, prostate cancer, risk reduction

## Abstract

Prostate cancer is among the leading causes of cancer-related morbidity and mortality in men worldwide. Despite advancements in diagnostic and therapeutic approaches, prevention remains a critical strategy to curb its prevalence. This review examines promising prevention practices, including lifestyle modifications, dietary interventions, and the use of chemopreventive agents. It also delves into emerging insights from genetic, epigenetic, and molecular studies, offering a comprehensive framework for reducing prostate cancer risk through tailored and evidence-based approaches. Lifestyle changes, such as maintaining a healthy weight, engaging in regular physical activity, and adopting a plant-based diet, have shown substantial potential in mitigating prostate cancer risk. Specific dietary components, like antioxidants found in fruits and vegetables and omega-3 fatty acids in fish, demonstrate protective properties, while reducing the intake of red and processed meats is encouraged. Chemopreventive agents, including 5-alpha reductase inhibitors and anti-inflammatory medications, offer additional strategies but require careful assessment of risks and benefits.

**HIGHLIGHTS**

- **Diet**: eat a diet rich in fruits, vegetables, and healthy fats.
- **Exercise**: regular physical activity lowers risk.
- **Healthy weight**: maintain a healthy weight.
- **Screening**: regular PSA tests and screenings.
- **Medication**: consider medications like finasteride for high-risk individuals.

## Introduction

Prostate cancer is one of the most prevalent malignancies affecting men globally and represents a major concern in cancer prevention and public health. According to the World Health Organization, it is the second most frequently diagnosed cancer in men and the fifth leading cause of cancer-related death worldwide. The burden is especially significant in high-income countries where routine screening and longer life expectancies contribute to higher detection rates. Despite advances in diagnosis and treatment, the disease continues to impose a considerable burden on healthcare systems, families, and affected individuals^[1,2]^. Traditionally, prostate cancer prevention has been approached through population-wide screening strategies, most notably prostate-specific antigen (PSA) testing. While this approach has contributed to early detection, it has also raised concerns regarding overdiagnosis, overtreatment, and psychological distress due to the identification of clinically insignificant tumors. Consequently, there is growing interest in refining preventive strategies to balance early detection with the avoidance of unnecessary intervention. This shift has brought prevention into sharper focus – not only through early screening but also through proactive efforts aimed at reducing the risk of disease onset^[3,4]^. A growing body of research has demonstrated that modifiable lifestyle factors such as diet, physical activity, and weight management can influence prostate cancer risk. Dietary components, particularly high consumption of red and processed meats, saturated fats, and low intake of fruits and vegetables, have been associated with increased risk. Conversely, diets rich in antioxidants, omega-3 fatty acids, and phytonutrients may offer protective benefits. These findings support the integration of nutritional and behavioral counseling into preventive healthcare services[5].

In parallel, pharmacologic approaches have emerged, particularly the use of 5-alpha-reductase inhibitors (5-ARIs) such as finasteride and dutasteride, which have demonstrated efficacy in reducing the incidence of prostate cancer in large clinical trials. However, these interventions are not without controversy. Some studies suggest a potential increase in the incidence of high-grade tumors among users, raising caution about the broad application of such chemopreventive agents. Nonetheless, they continue to represent a promising avenue, especially for individuals with elevated baseline risk^[6,7]^. Nutraceuticals and dietary supplements, including selenium, vitamin E, lycopene, and green tea catechins, have also been studied extensively for their potential chemopreventive roperties. Despite encouraging results from laboratory and observational studies, randomized controlled trials have yielded inconsistent or even contradictory findings. For instance, the Selenium and Vitamin E Cancer Prevention Trial (SELECT) found no benefit – and even potential harm – from these supplements, highlighting the complexity of nutrient-based interventions in cancer prevention[8]. Another emerging dimension in prostate cancer prevention is genetic and molecular profiling. As scientific understanding of hereditary risk and genetic mutations deepens, tools such as polygenic risk scores and genomic sequencing are being explored to personalize prevention. These innovations could revolutionize risk stratification and inform tailored screening and intervention strategies, particularly for high-risk groups such as men with a family history or those of African descent, who bear a disproportionate burden of prostate cancer mortality[9]. Moreover, the advent of precision medicine and digital health technologies has ushered in new possibilities for prevention. Artificial intelligence (AI)-assisted risk prediction models, mobile health apps, and remote monitoring systems are being integrated into prostate cancer prevention research. These innovations may offer scalable, cost-effective ways to engage men in risk reduction practices while enhancing the accuracy of early detection and individualized decision-making[10].

## Aim

The aim of this comprehensive review is to provide a thorough exploration of prostate cancer risk reduction strategies, encompassing both conventional and integrative approaches.

### Review methods

This narrative review was conducted to synthesize current evidence on promising practices and emerging trends in the prevention of prostate cancer. Although narrative reviews do not follow the rigid protocol of systematic reviews, a structured and transparent approach was applied in the literature search, study selection, and synthesis of findings.

### Databases searched

To ensure comprehensive coverage of relevant literature, multiple electronic databases were searched. These included PubMed, Scopus, Web of Science, Google Scholar, and the Cochrane Library. The search was carried out between January 2005 and 31 March 2025, to include the most recent advances in the field of prostate cancer prevention. Earlier landmark studies were also included when deemed foundational.

### Search terms used

A combination of Medical Subject Headings (MeSH) and free-text terms were used to retrieve relevant articles. The following search terms and Boolean operators were employed:

- (“prostate cancer “or” prostatic neoplasms”) and
- (“prevention “or” risk reduction “or” chemoprevention”) and
- (“lifestyle modification “or” diet “or” exercise “or” weight control”) and
- (“pharmacological “or” 5-alpha reductase inhibitors “or” finasteride “or” dutasteride”) and
- (“screening “or” psa testing “or” early detection”) and
- (“genetics “or” brca “or” polygenic risk scores”) and
- (“immunoprevention “or” nanotechnology “or” biomarkers”).

Search strategies were tailored to suit the indexing and capabilities of each database. Citation tracking and reference list reviews were also performed to identify additional relevant studies.

### Inclusion criteria

- Studies were included in the review based on the following criteria:
- Peer-reviewed articles published in English.
- Studies focusing on human subjects.
- Articles presenting primary data, reviews, or meta-analyses related to prostate cancer prevention.

- Studies addressing one or more preventive strategies, including lifestyle, pharmacological interventions, genetic risk assessments, screening innovations, or emerging technologies.
- Studies involving high-risk populations or providing new insights into preventive mechanisms.

### Exclusion criteria

The following studies were excluded:

- Non-English language articles.
- Studies focusing solely on prostate cancer treatment without preventive implications.
- Editorials, opinion pieces, and conference abstracts without full data.
- Animal studies unless directly translatable to human prevention.
- Studies with incomplete data or lacking peer-review.

### Study selection

All identified citations were exported into reference management software (Zotero). Titles and abstracts were screened independently by two reviewers. Full-text articles were retrieved for potentially eligible studies and assessed for final inclusion based on the criteria listed above. Discrepancies in article selection were resolved through discussion and consensus. We initially retrieved 120 articles. After screening titles and abstracts and applying eligibility criteria, 51 articles were included in the final synthesis.

### Quality assessment tools

Although the nature of a narrative review does not mandate formal risk-of-bias evaluation, an informal quality assessment was conducted to ensure the credibility and relevance of included studies. The Critical Appraisal Skills Programme (CASP) tools were used to evaluate systematic reviews and cohort studies, while the AMSTAR 2 checklist was referenced for assessing meta-analyses. Particular attention was paid to sample size, methodological clarity, relevance to prevention, and strength of conclusions. Only studies meeting a moderate to high level of quality were included in the synthesis. This structured yet flexible approach ensured that the evidence base used in this narrative review was robust, current, and reflective of both established practices and innovative developments in prostate cancer prevention.

### Overview of risk factors associated with prostate cancer

Prostate cancer development is influenced by a complex interplay of genetic, environmental, and lifestyle factors.

### Non-modifiable risk factors

1. **Age**

Age is the strongest risk factor for prostate cancer. The likelihood of developing prostate cancer increases significantly after the age of 50, with the majority of cases diagnosed in men over 65 years. This correlation underscores the importance of regular screening and early detection in older populations[11].

1. **Genetic Predisposition**

Family history plays a critical role in prostate cancer risk. Men with a first-degree relative (father or brother) diagnosed with prostate cancer have a two- to three-fold increased risk of developing the disease. Mutations in specific genes, such as **BRCA1, BRCA2**, and **HOXB13**, have been identified as contributors to hereditary prostate cancer. These genetic markers provide valuable insights into individual susceptibility and highlight the need for genetic counseling in high-risk families[12].

1. **Ethnicity**

Ethnic background significantly influences prostate cancer risk. African American men have the highest incidence and mortality rates compared to men of other ethnic groups. Conversely, Asian and Hispanic men tend to have lower rates. These disparities may be due to genetic, environmental, and healthcare access factors, emphasizing the need for targeted prevention strategies[13].

### Modifiable risk factors

1. **Diet**

Dietary habits play a significant role in prostate cancer risk modulation. Diets high in saturated fats, red and processed meats, and dairy products have been linked to an increased risk, while plant-based diets rich in fruits, vegetables, and whole grains are associated with a protective effect. Specific components, such as lycopene in tomatoes, selenium, and omega-3 fatty acids, have shown promise in reducing risk[14].

1. **Obesity and Body Weight**

Obesity is associated with an increased risk of aggressive prostate cancer. Excess body fat contributes to systemic inflammation, hormonal imbalances, and metabolic changes that may promote cancer progression. Maintaining a healthy weight through balanced nutrition and regular exercise is an essential preventive measure.

1. **Physical**[15] **Activity**

Regular physical activity has been linked to a lower risk of prostate cancer and improved outcomes for those diagnosed. Exercise reduces inflammation, modulates hormone levels, and enhances immune function, collectively contributing to its protective effects[16].

1. **Smoking and Alcohol Consumption**

Although smoking is not a primary risk factor for prostate cancer, it has been linked to higher mortality and recurrence rates among affected individuals. Excessive alcohol consumption may also contribute indirectly by exacerbating other risk factors, such as obesity and inflammation[17].

1. **Hormonal Factors**

Hormonal imbalances, particularly elevated levels of androgens, are associated with increased prostate cancer risk. Understanding these hormonal mechanisms is critical for developing targeted prevention strategies, such as the use of androgen receptor inhibitors.

### Emerging risk factors

1. **Epigenetic Modifications**

Research indicates that epigenetic changes, including DNA methylation and histone modification, may influence prostate cancer development. These alterations can be affected by lifestyle and environmental factors, offering potential avenues for risk reduction through interventions[15].

1. **Gut Microbiome**

The gut microbiome is emerging as a significant modulator of prostate cancer risk. Dysbiosis, or an imbalance in gut microbial populations, may contribute to systemic inflammation and hormonal changes, both of which are implicated in cancer progression[16].

1. **Chronic Inflammation**

Chronic inflammation in the prostate, often due to infections or autoimmune conditions, has been linked to an increased risk of cancer. Understanding the role of inflammation in prostate carcinogenesis may pave the way for preventive strategies targeting immune modulation[17].

### Physical activity

Physical activity is increasingly recognized as a vital component in reducing the risk of prostate cancer and improving outcomes for those already diagnosed. Regular exercise has multifaceted benefits that extend beyond general health maintenance, directly influencing cancer risk and progression through several biological mechanisms.

#### Protective mechanisms of physical activity

1. **Hormonal Modulation**

Physical activity helps regulate levels of hormones, such as insulin, androgens, and growth factors that play a role in prostate cancer development. Elevated insulin-like growth factor-1 (IGF-1), for instance, has been associated with increased prostate cancer risk. Regular exercise reduces IGF-1 levels and improves insulin sensitivity, thereby mitigating this risk[15].

1. **Reduction of Systemic Inflammation**

Chronic inflammation is a known contributor to cancer initiation and progression. Exercise reduces inflammatory markers, such as C-reactive protein (CRP) and interleukin-6 (IL-6), creating an anti-inflammatory environment that may lower the risk of prostate cancer[16].

1. **Enhanced Immune Surveillance**

Physical activity boosts immune function by increasing the circulation of natural killer (NK) cells, T-cells, and other immune components. Enhanced immune surveillance helps the body detect and eliminate abnormal cells before they progress to malignancy[16].

1. **Weight Management**

Exercise plays a critical role in maintaining a healthy weight, which is closely linked to reduced prostate cancer risk. Obesity is associated with more aggressive forms of prostate cancer, and physical activity aids in mitigating this risk by reducing body fat and improving metabolic health[17].

#### Types and intensity of physical activity

1. **Aerobic Exercise**

Activities such as walking, jogging, cycling, and swimming are effective in reducing prostate cancer risk. Aerobic exercises improve cardiovascular health, regulate hormone levels, and reduce systemic inflammation[15].

1. **Resistance Training**

Strength training exercises, including weightlifting and resistance band workouts, are beneficial for maintaining muscle mass and metabolic health. They complement aerobic activities by improving overall body composition and physical resilience[16].

1. **Moderate to Vigorous Intensity**

The intensity and duration of physical activity matter. Studies indicate that moderate to vigorous physical activity, sustained for at least 150 minutes per week, yields the most significant protective benefits against prostate cancer[17].

#### Physical activity for prostate cancer survivors

For men diagnosed with prostate cancer, physical activity improves treatment outcomes and quality of life. Exercise has been shown to alleviate treatment-related side effects, such as fatigue, depression, and urinary incontinence. Moreover, it helps mitigate the long-term risks of cancer recurrence and progression by promoting a healthier systemic environment. Despite the benefits, barriers such as lack of time, physical limitations, or lack of awareness may hinder adherence to regular physical activity. Healthcare providers play a critical role in promoting exercise as part of a preventive and therapeutic strategy. Tailored exercise programs, considering individual fitness levels and health status, can maximize adherence and effectiveness[17].

### Smoking cessation

Smoking has long been associated with a range of adverse health outcomes, including cardiovascular disease, respiratory illnesses, and various cancers. While its direct link to prostate cancer risk is less pronounced than for lung or oral cancers, smoking has been implicated in the progression of aggressive prostate cancer and poorer treatment outcomes. Consequently, smoking cessation is a critical preventive measure for individuals seeking to lower their overall cancer risk, including prostate cancer[18]. Smokers are more likely to develop aggressive forms of prostate cancer compared to non-smokers. Tobacco contains carcinogenic compounds that can cause DNA damage, induce oxidative stress, and promote systemic inflammation – all of which contribute to cancer initiation and progression. Additionally, smoking may alter hormone levels, such as testosterone and insulin-like growth factors, which are implicated in prostate carcinogenesis. More importantly, smoking has been linked to poorer outcomes in prostate cancer patients. Studies show that smokers are at a higher risk of biochemical recurrence following prostatectomy and are less likely to respond favorably to radiation therapy. The presence of smoking-related comorbidities, such as cardiovascular disease, further complicates treatment and diminishes overall survival rates[19].

The benefits of smoking cessation for prostate cancer prevention and management are significant. Quitting smoking reduces systemic inflammation and oxidative stress, creating a less favorable environment for cancer growth. Research suggests that individuals who quit smoking for ten years or more have a prostate cancer risk comparable to that of never-smokers, underscoring the long-term benefits of cessation. For prostate cancer patients, smoking cessation enhances treatment efficacy and reduces the likelihood of recurrence. For instance, individuals who quit smoking before undergoing surgery or radiation therapy show better postoperative recovery and improved survival rates. Additionally, cessation mitigates treatment side effects, such as cardiovascular complications and pulmonary dysfunction, which are exacerbated by smoking[20]. While the benefits of smoking cessation are clear, achieving long-term abstinence remains a challenge for many individuals. Nicotine addiction, stress, and social influences often hinder the cessation process. However, evidence-based interventions can significantly improve success rates. Behavioral counseling, either one-on-one or in group settings, helps individuals develop coping mechanisms and strategies to resist smoking triggers. Pharmacological aids, such as nicotine replacement therapy (NRT), bupropion, and varenicline, are effective in reducing withdrawal symptoms and cravings. Integrating these approaches into a comprehensive smoking cessation program maximizes the likelihood of sustained success[19]. At the societal level, public health initiatives play a vital role in reducing smoking prevalence. Policies such as increased tobacco taxes, restrictions on smoking in public spaces, and mass media campaigns have proven effective in discouraging smoking. For men at risk of prostate cancer, targeted awareness campaigns that highlight the link between smoking and cancer outcomes can motivate individuals to quit[20].

### Nutritional strategies

#### Diet

Diet plays a significant role in prostate cancer prevention, with numerous studies indicating that certain dietary patterns and specific foods can influence the risk of developing prostate cancer[21]. Adopting a balanced diet rich in fruits, vegetables, whole grains, and healthy fats can contribute to reducing the incidence of prostate cancer and improving overall health. A diet high in fruits and vegetables is associated with a lower risk of prostate cancer[22]. These foods are rich in vitamins, minerals, and antioxidants, which help protect cells from damage. Specific vegetables, such as tomatoes, broccoli, and other cruciferous vegetables, have been studied for their potential protective effects. Tomatoes, in particular, contain lycopene, an antioxidant that has been linked to a reduced risk of prostate cancer. Cruciferous vegetables, like broccoli, cauliflower, and Brussels sprouts, contain sulforaphane and other compounds that may help prevent cancer development. Whole grains, such as oats, brown rice, and whole wheat, are an essential part of a prostate-healthy diet[22]. These grains are high in fiber, which helps regulate digestion and may reduce the risk of developing prostate cancer. Fiber also helps maintain a healthy weight, which is important since obesity is a known risk factor for aggressive prostate cancer. Additionally, whole grains provide essential nutrients that support overall health. Incorporating healthy fats into the diet is crucial for prostate cancer prevention. Omega-3 fatty acids, found in fatty fishlike salmon, mackerel, and sardines, have anti-inflammatory properties that can help reduce cancer risk. These fats may also slow the growth of prostate cancer cells. Conversely, it is advisable to limit the intake of saturated fats and trans fats, commonly found in red and processed meats, full-fat dairy products, and fried foods, as they have been associated with an increased risk of prostate cancer.

Reducing the consumption of red and processed meats is recommended for prostate cancer prevention. Studies have shown that diets high in red meat, particularly when cooked at high temperatures, can increase the risk of prostate cancer^[23,24]^. Processed meats, such as sausages, bacon, and deli meats, contain nitrates and other preservatives that are carcinogenic. Opting for plant-based protein sources, such as beans, lentils, and tofu, or lean meats like poultry and fish, can help lower cancer risk. The relationship between dairy products and prostate cancer risk is complex and somewhat controversial. Some studies suggest that high consumption of dairy products, particularly those high in fat, may be linked to an increased risk of prostate cancer. This is thought to be due to the high levels of calcium and hormones found in dairy. However, more research is needed to fully understand this relationship. Moderation and choosing low-fat or fat-free dairy options may be prudent for those concerned about prostate cancer risk. Foods rich in antioxidants and phytochemicals are particularly beneficial in cancer prevention. These compounds help neutralize harmful free radicals in the body, reducing oxidative stress and inflammation. Berries, nuts, seeds, green tea, and dark chocolate are excellent sources of antioxidants. Including a variety of these foods in the diet can provide a broad spectrum of protective compounds[23]. Soy products, such as tofu, tempeh, and edamame, contain phytoestrogens that may have protective effects against prostate cancer. These compounds can help regulate hormone levels and potentially reduce the growth of cancer cells. Populations with high soy consumption, such as those in Asian countries, tend to have lower rates of prostate cancer, suggesting a potential protective role of soy in the diet[24].

The Mediterranean diet, characterized by high consumption of fruits, vegetables, whole grains, nuts, seeds, olive oil, and moderate intake of fish and poultry, has been associated with numerous health benefits, including a reduced risk of prostate cancer. This diet is rich in healthy fats, fiber, and antioxidants, which collectively contribute to cancer prevention. Adopting a Mediterranean-style diet can be an effective strategy for reducing prostate cancer risk and promoting overall health. Extensive research supports the role of diet in prostate cancer prevention. A study published in the *Journal of the National Cancer Institute* found that a diet high in fruits and vegetables and low in animal fats was associated with a reduced risk of prostate cancer. Another study in *Cancer Epidemiology, Biomarkers & Prevention* highlighted the protective effects of a Mediterranean diet against prostate cancer^[25,26]^. These findings underscore the importance of dietary choices in cancer prevention strategies.

#### Supplements

The use of dietary supplements for prostate cancer prevention is a subject of ongoing research and debate. While certain supplements have shown potential in reducing the risk of prostate cancer, others have been associated with either no benefit or even potential harm. It is crucial to approach supplementation with caution and to consider current evidence when incorporating them into a prostate cancer prevention strategy. Vitamin E, an antioxidant, was once thought to have cancer-preventive properties. However, large-scale studies, such as the Selenium and Vitamin E Cancer Prevention Trial (SELECT), found that high-dose vitamin E supplementation actually increased the risk of prostate cancer[27]. These findings highlight the importance of evidence-based supplementation and caution against high-dose vitamin E supplements. Selenium is another supplement that has been extensively studied for its potential role in prostate cancer prevention[28]. Initial studies suggested that selenium might reduce prostate cancer risk, but subsequent research, including the SELECT trial, found no protective benefit and raised concerns about potential risks. Selenium supplementation should be approached cautiously, and it is generally recommended to obtain selenium through a balanced diet rather than supplements. Lycopene, a carotenoid found in tomatoes and other red fruits and vegetables, has shown promise in reducing prostate cancer risk.30 Lycopene is a potent antioxidant that helps protect cells from damage. Several studies have suggested that higher dietary intake of lycopene is associated with a lower risk of prostate cancer. While lycopene supplements are available, consuming lycopene-rich foods is generally preferred for obtaining its benefits.

Omega-3 fatty acids, found in fish oil and flaxseed oil, have anti-inflammatory properties that may help reduce prostate cancer risk. Some studies have shown a potential protective effect of omega-3s against prostate cancer, while others have found no significant benefit^[29,30]^. It is generally recommended to obtain omega-3s through dietary sources, such as fatty fish, rather than relying solely on supplements. Green tea contains polyphenols, particularly epigallocatechin gallate (EGCG), which have been studied for their anti-cancer properties. Some research suggests that green tea and its extracts may help reduce the risk of prostate cancer. Regular consumption of green tea is considered beneficial, and green tea supplements may also offer some protective effects, though more research is needed to confirm their efficacy[29]. Zinc is an essential mineral that plays a role in immune function and cell growth. Excessive zinc supplementation can have adverse effects, so it is important to maintain a balanced intake through diet and supplements, if necessary, under medical supervision. The use of multivitamins for prostate cancer prevention is controversial. Some studies suggest that regular multivitamin use may reduce the risk of certain cancers, while others indicate no benefit or even potential harm. It is important to approach multivitamin use with caution and to choose formulations that do not exceed recommended daily allowances of vitamins and minerals. Various herbal supplements, such as saw palmetto and pygeum, are commonly used for prostate health. While these supplements are often marketed for benign prostatic hyperplasia (BPH), their efficacy in prostate cancer prevention is not well established. More rigorous clinical trials are needed to determine their potential role in reducing prostate cancer risk[30]. Probiotics, beneficial bacteria that support gut health, have been studied for their potential role in cancer prevention. Some research suggests that probiotics may help modulate the immune system and reduce inflammation, potentially lowering prostate cancer risk. Including probiotic-rich foods, such as yogurt and fermented vegetables, in the diet can be beneficial, and probiotic supplements may also offer some advantages.

#### Chemoprevention

Chemoprevention refers to the use of natural or synthetic agents to prevent the development or progression of cancer. In the context of prostate cancer, several agents have shown promise in reducing risk. These include 5-alpha reductase inhibitors, selective estrogen receptor modulators (SERMs), nonsteroidal anti-inflammatory drugs (NSAIDs), and other compounds. This section explores the potential and challenges of chemoprevention in prostate cancer.5-alpha reductase inhibitors, such as finasteride and dutasteride, have been extensively studied for their role in prostate cancer prevention[31]. These medications work by blocking the conversion of testosterone to dihydrotestosterone (DHT), a hormone that stimulates prostate growth. The Prostate Cancer Prevention Trial (PCPT) and the Reduction by Dutasteride of Prostate Cancer Events (REDUCE) trial demonstrated that these drugs could reduce the incidence of prostate cancer, particularly lower-grade tumors. In the PCPT, finasteride reduced the overall risk of prostate cancer by about 25%. However, there was an observed increase in the incidence of high-grade tumors, raising concerns about the drug’s safety profile. Subsequent analysis suggested that finasteride might make it easier to detect high-grade cancers due to its effect on prostate size and PSA levels rather than causing the cancers directly. The REDUCE trial showed similar results with dutasteride, with a 23% reduction in prostate cancer risk. Like finasteride, dutasteride was associated with a higher detection rate of high-grade tumors, but the overall impact on mortality remains unclear.

SERMs, such as toremifene, have also been investigated for prostate cancer prevention[32]. These compounds selectively modulate estrogen receptors, exhibiting both estrogenic and anti-estrogenic effects depending on the tissue. Toremifene has shown potential in reducing the risk of prostate cancer in men with high-grade prostatic intraepithelial neoplasia (PIN), a precursor to prostate cancer. Clinical trials have demonstrated that toremifene can decrease the incidence of prostate cancer in men with high-grade PIN. However, the long-term benefits and potential side effects require further study to establish its role in routine chemoprevention. NSAIDs, including aspirin and other anti-inflammatory drugs, have been studied for their potential to reduce prostate cancer risk[33]. These drugs inhibit cyclooxygenase (COX) enzymes, which play a role in inflammation and cancer development. Some observational studies suggest that regular aspirin use may be associated with a reduced risk of prostate cancer, particularly advanced forms of the disease. However, the evidence is not consistent, and the potential risks, such as gastrointestinal bleeding, must be weighed against the benefits. The use of other NSAIDs for prostate cancer prevention is less well studied. While they may offer some protective effects, the risk of adverse effects, including cardiovascular events and gastrointestinal issues, limits their routine use for chemoprevention. Statins, commonly used to lower cholesterol, have been explored for their potential anti-cancer properties. Some studies suggest that statins may reduce the risk of advanced prostate cancer, possibly due to their anti-inflammatory and cell growth-regulatory effects. Research indicates that statins may reduce prostate cancer risk by lowering cholesterol levels and affecting molecular pathways involved in cancer cell proliferation. However, the evidence is mixed, and further studies are needed to clarify their role in prostate cancer prevention^[34,35]^. Various natural compounds and phytochemicals, such as curcumin, green tea catechins, and lycopene, have been studied for their chemopreventive properties. These agents exhibit antioxidant, anti-inflammatory, and anti-carcinogenic effects, making them promising candidates for prostate cancer prevention. Derived from turmeric, curcumin has shown potential in preclinical studies for inhibiting prostate cancer cell growth and inducing apoptosis. Clinical trials are ongoing to evaluate its efficacy and safety in humans. Compounds found in green tea, particularly EGCG, have demonstrated anti-cancer properties in laboratory studies. Some clinical trials suggest that green tea extract may reduce the risk of prostate cancer in high-risk individuals. As mentioned earlier, lycopene is a potent antioxidant found in tomatoes and other red fruits. Its potential role in chemoprevention is supported by studies showing lower prostate cancer rates in populations with high dietary lycopene intake.

#### Genetic factors

Genetic factors play a significant role in prostate cancer risk, with certain inherited genetic mutations and family history contributing to an increased likelihood of developing the disease. A family history of prostate cancer is one of the most well-established risk factors[36]. Men with a first-degree relative (father or brother) who has had prostate cancer are more than twice as likely to develop the disease themselves. The risk increases further if multiple relatives are affected or if the family history includes early-onset prostate cancer. Several specific genetic mutations have been identified that increase the risk of prostate cancer. These include mutations in the BRCA1 and BRCA2 genes, which are also associated with breast and ovarian cancers. Men with BRCA2 mutations, in particular, have a significantly elevated risk of developing prostate cancer, often at a younger age and with more aggressive forms of the disease. Mutations in these genes impair DNA repair mechanisms, leading to an increased likelihood of cancer development. Men with BRCA2 mutations face a higher risk compared to those with BRCA1 mutations. Genetic testing for these mutations can help identify individuals at increased risk and guide preventive measures. Another gene associated with prostate cancer is HOXB13. Mutations in this gene have been linked to hereditary prostate cancer, particularly in families with a strong history of the disease. The G84E mutation in HOXB13 has been identified as a significant risk factor for early-onset prostate cancer.

Lynch syndrome, also known as hereditary nonpolyposis colorectal cancer (HNPCC), is associated with a higher risk of several cancers, including prostate cancer[37]. Men with Lynch syndrome have mutations in mismatch repair (MMR) genes, such as MLH1, MSH2, MSH6, and PMS2, which lead to increased cancer risk due to impaired DNA repair.In addition to specific mutations, various genetic polymorphisms (common genetic variations) have been linked to prostate cancer risk. Genome-wide association studies (GWAS) have identified numerous single nucleotide polymorphisms (SNPs) associated with increased susceptibility to prostate cancer. These polymorphisms can be used to develop polygenic risk scores, which estimate an individual’s genetic risk based on the combined effect of multiple genetic variants. Polygenic risk scores integrate information from multiple genetic variants to provide an overall assessment of genetic risk for prostate cancer. These scores can help stratify individuals into different risk categories, potentially guiding screening and preventive strategies. Men with high polygenic risk scores may benefit from more intensive monitoring and early intervention.

Genetic testing for prostate cancer risk involves analyzing specific genes and genetic variants associated with the disease[38]. Testing can be particularly beneficial for men with a strong family history or other risk factors. Genetic counseling is an essential component of the testing process, helping individuals understand their risk and the implications of test results. Identifying high-risk individuals through genetic testing allows for personalized preventive measures, including increased surveillance and consideration of risk-reducing interventions. For example, men with BRCA2 mutations may benefit from more frequent prostate-specific antigen (PSA) testing and early biopsy. Genetic testing also raises ethical and psychological considerations. Individuals must be informed about the potential implications of test results, including the impact on family members and the possibility of genetic discrimination. Genetic counseling provides support in navigating these complex issues[38]. High-risk individuals identified through genetic testing can benefit from tailored screening programs and preventive strategies. This may include earlier and more frequent PSA testing, magnetic resonance imaging (MRI) scans, and consideration of chemoprevention or prophylactic treatments. For men with a significant genetic risk, screening recommendations may differ from the general population. Early and more frequent PSA testing, starting at age 40 or even earlier for those with known high-risk mutations, can help detect prostate cancer at a more treatable stage. Preventive interventions for high-risk individuals may include lifestyle modifications, chemoprevention, and consideration of surgical options in select cases. Ongoing research aims to develop more effective strategies tailored to the genetic risk profile of each individual.

#### Hormonal interventions

Hormonal interventions are a significant aspect of prostate cancer management, particularly in cases where the cancer is hormone-sensitive or advanced. These interventions target the male hormones (androgens) that fuel prostate cancer growth, aiming to either reduce hormone levels or block their effects on cancer cells. This section explores various hormonal interventions used in prostate cancer treatment and their role in improving outcomes. Androgen deprivation therapy (ADT), also known as hormone therapy, is the cornerstone of treatment for advanced or metastatic prostate cancer[39]. ADT aims to lower the levels of male hormones, particularly testosterone, which fuels prostate cancer cell growth. ADT can be achieved through surgical removal of the testicles (orchiectomy) or, more commonly, by using medications that suppress testosterone production or block its action. These medications include luteinizing hormone-releasing hormone (LHRH) agonists (such as leuprolide and goserelin) and antagonists (such as degarelix). ADT is often the initial treatment for metastatic prostate cancer, aiming to shrink tumors and slow disease progression. ADT may be used in combination with radiation therapy for locally advanced disease to enhance treatment effectiveness. In cases of rising PSA levels after primary treatment (such as surgery or radiation), ADT may be used to delay disease progression. ADT can cause significant side effects due to the reduction in testosterone levels, including hot flashes, loss of libido, erectile dysfunction, fatigue, and osteoporosis. Long-term use of ADT may also increase the risk of cardiovascular disease and metabolic disorders.

Anti-androgens are medications that block the effects of androgens on prostate cancer cells[40]. They are often used in combination with ADT or as monotherapy in cases where ADT alone is not sufficient. Anti-androgens are commonly used in combination with LHRH agonists or antagonists to achieve maximal androgen blockade in advanced prostate cancer. They may also be used in intermittent ADT regimens to mitigate side effects. Side effects of anti-androgen therapy may include fatigue, nausea, liver toxicity (more common with non-steroidal agents), and occasionally, breast tenderness or gynecomastia (enlargement of breast tissue). In cases where prostate cancer progresses despite initial ADT, second-line hormonal therapies may be considered to further suppress androgen receptor signaling or production. Drugs such as enzalutamide and abiraterone acetate target the androgen receptor pathway at different points. Enzalutamide is an androgen receptor inhibitor that blocks androgen binding to the receptor, while abiraterone acetate inhibits the enzyme (CYP17) necessary for androgen synthesis in the adrenal glands and tumor tissue. Second-line hormonal therapies are used in metastatic castration-resistant prostate cancer (mCRPC), where cancer continues to progress despite low testosterone levels achieved with ADT. Common side effects of second-line hormonal therapies include fatigue, hypertension (with abiraterone), electrolyte imbalances (with abiraterone), and potentially increased risk of cardiovascular events. Estrogen therapy, using medications like diethylstilbestrol (DES), was historically used for prostate cancer before the advent of ADT and anti-androgen therapies. Estrogens suppress testosterone production by the testes and were effective in reducing symptoms and tumor size in some cases. Estrogen therapy is less commonly used today due to the availability of more effective and safer options like ADT and anti-androgen therapies. It is reserved for cases where other treatments have failed or are contraindicated. Estrogen therapy can cause cardiovascular complications, including venous thromboembolism (blood clots), fluid retention, breast tenderness, and potentially feminizing effects[40].

### Integrative and complementary approaches

Integrative and complementary approaches are gaining attention in prostate cancer care, offering supportive strategies alongside conventional treatments to enhance quality of life, manage side effects, and potentially improve treatment outcomes[41]. These approaches encompass lifestyle modifications, mind-body practices, dietary supplements, and alternative therapies, which may be used in conjunction with standard medical treatments. Regular physical activity is beneficial for overall health and can help manage side effects of prostate cancer treatment, such as fatigue and muscle weakness. Exercise may also improve mood and quality of life. Recommendations often include aerobic exercises, strength training, and flexibility exercises tailored to individual abilities and preferences. Adopting a balanced diet rich in fruits, vegetables, whole grains, and lean proteins can support overall health and potentially reduce inflammation. Specific dietary components, such as lycopene from tomatoes and omega-3 fatty acids from fish, have been studied for their potential protective effects against prostate cancer. Quitting smoking is crucial for prostate cancer patients, as smoking is linked to a higher risk of aggressive prostate cancer and can complicate treatment outcomes.

**Mindfulness-Based Stress Reduction (MBSR)** techniques, including meditation and mindful breathing exercises, can help reduce stress, anxiety, and depression commonly experienced by prostate cancer patients[41]. These practices may improve overall well-being and coping mechanisms during treatment. These mind-body practices combine physical movements, breathing exercises, and meditation. They can enhance flexibility, balance, and relaxation, potentially reducing treatment-related symptoms such as fatigue and improving quality of life. Adequate levels of vitamin D may support immune function and bone health in prostate cancer patients. Vitamin D supplementation is often recommended, especially for individuals with low levels. Rich in antioxidants like polyphenols, green tea extract has shown potential in preclinical studies for its anti-cancer properties. It may complement standard treatments by reducing oxidative stress and inflammation. Derived from turmeric, curcumin has anti-inflammatory and antioxidant properties. Studies suggest it may inhibit prostate cancer cell growth and enhance the effects of chemotherapy or radiation therapy. Acupuncture involves inserting thin needles into specific points on the body to alleviate pain and promote healing. It may help manage treatment-related side effects such as pain, nausea, and fatigue. Traditional Chinese herbal formulations are sometimes used in conjunction with conventional treatments to support immune function, reduce inflammation, and improve overall well-being. However, their use should be supervised by qualified practitioners familiar with oncology care. Massage can relieve muscle tension, improve circulation, and promote relaxation. It may help alleviate pain and discomfort associated with prostate cancer treatment. Physical therapists can provide exercises and techniques to improve mobility, strength, and function, particularly important for patients recovering from surgery or managing treatment-related side effects. Joining support groups or counseling sessions can provide emotional support, practical advice, and a sense of community for prostate cancer patients and their caregivers. Professional counseling can help patients and their families cope with the emotional impact of a prostate cancer diagnosis, treatment decisions, and survivorship issues. Integrative oncology programs combine conventional treatments with complementary therapies in a coordinated approach. These programs often involve collaboration among oncologists, integrative medicine specialists, nutritionists, and other healthcare providers. Integrative oncology programs prioritize evidence-based practices and emphasize safety, efficacy, and patient-centered care. They may offer personalized treatment plans tailored to individual needs and preferences[42].

### Environmental factors

Environmental factors play a complex and evolving role in prostate cancer risk, influencing both its development and progression[43]. Agricultural workers and individuals exposed to pesticides and herbicides, such as organochlorines (e.g., DDT) and organophosphates, may face an increased risk of prostate cancer. These chemicals have been linked to endocrine disruption and carcinogenesis, although the evidence remains inconclusive and subject to ongoing research. Exposure to heavy metals like cadmium, arsenic, and lead through occupational settings or environmental pollution may contribute to prostate cancer risk. These metals can accumulate in the body over time, potentially disrupting cellular functions and promoting cancer development. **Polycyclic Aromatic Hydrocarbons (PAHs)** are environmental pollutants released during the incomplete combustion of organic substances, such as coal, oil, and tobacco. Occupational exposure to PAHs, as well as exposure through air pollution and tobacco smoke, has been associated with an increased risk of prostate cancer. Diets high in red meat, processed foods, and saturated fats may increase prostate cancer risk. Conversely, diets rich in fruits, vegetables, and fish containing omega-3 fatty acids are associated with a lower risk. The balance of nutrients, antioxidants, and phytochemicals in the diet plays a critical role in prostate cancer prevention. Obesity and excess body fat are linked to an increased risk of aggressive prostate cancer and poorer treatment outcomes. Adipose tissue produces hormones and cytokines that can promote cancer growth and progression, highlighting the importance of maintaining a healthy weight. Regular physical activity is associated with a reduced risk of developing aggressive prostate cancer. Exercise helps regulate hormone levels, reduce inflammation, and enhance immune function, contributing to overall cancer prevention. Smoking tobacco is linked to an increased risk of aggressive prostate cancer and poorer treatment outcomes. The carcinogens in tobacco smoke may directly affect prostate tissue and contribute to cancer development.

Certain occupations involving exposure to carcinogens, such as firefighters exposed to combustion by-products and industrial workers exposed to chemicals, may face an increased risk of prostate cancer[44]. Occupational safety measures and protective equipment are crucial in mitigating these risks. Environmental chemicals known as endocrine disruptors, including bisphenol A (BPA) and phthalates, can interfere with hormonal balance and potentially contribute to prostate cancer risk. These chemicals are found in plastics, personal care products, and various industrial materials. Socioeconomic factors, including access to healthcare, education, and environmental quality, can influence prostate cancer risk and outcomes. Disparities in screening, diagnosis, and treatment access may contribute to poorer outcomes in certain populations. Prostate cancer incidence and mortality rates vary geographically, influenced by environmental factors such as pollution levels, dietary patterns, and lifestyle choices. Regional differences in healthcare infrastructure and screening practices also impact outcomes.

### Promising prevention practices for prostate cancer

Reducing the risk of prostate cancer involves a multifaceted approach that incorporates lifestyle modifications, dietary adjustments, regular screenings, and emerging preventive interventions. This section delineates several promising practices that individuals can adopt to potentially lower their risk of developing prostate cancer.

1. **Healthy Diet and Nutrition**

Emphasize a plant-based diet rich in fruits, vegetables, whole grains, and legumes. These foods contain antioxidants, vitamins, and phytochemicals that may confer protective benefits against prostate cancer. Limit the consumption of red and processed meats, as their high intake has been linked to an increased risk of prostate cancer[45]. Instead, opt for lean protein sources like fish, poultry, and plant-based proteins. Consider incorporating foods rich in omega-3 fatty acids, such as salmon, flaxseeds, and walnuts, which may have potential protective effects against prostate cancer.

1. **Regular Exercise and Weight Management**

Engage in regular physical activity, aiming for at least 150 minutes of moderate-intensity exercise per week. Exercise has been associated with a reduced risk of aggressive prostate cancer. Maintain a healthy weight and body mass index (BMI). Obesity and excess body fat, especially around the abdomen, have been linked to an increased risk of developing aggressive forms of prostate cancer^[46^-^48]^.

1. **Screening and Early Detection**

Adhere to recommended screening guidelines for prostate cancer, typically involving prostate-specific antigen (PSA) blood tests and digital rectal examinations (DRE)[46]. Discuss with a healthcare professional about the appropriate screening schedule based on individual risk factors and age. Early detection through routine screenings enables timely intervention and management, potentially improving treatment outcomes^[49,50]^.

1. Lifestyle Modifications

Avoid or limit tobacco use and excessive alcohol consumption. Smoking and heavy alcohol intake have been associated with an increased risk of aggressive prostate cancer[47]. Manage stress effectively through relaxation techniques, mindfulness practices, or seeking support from counselors or support groups. Chronic stress may negatively impact overall health and potentially influence cancer risk[51].

1. **Stay Informed and Participate in Research**

Stay informed about advancements in prostate cancer research, treatment options, and risk reduction strategies. Consider participating in clinical trials or studies that explore new preventive measures or treatments for prostate cancer. Contributing to research endeavors can advance the understanding of prostate cancer and its prevention.

## Discussion of conflicting findings: supplements and chemoprevention

The role of supplements and chemoprevention in prostate cancer prevention has been a subject of considerable debate, with conflicting findings emerging from various studies. Some studies suggest that certain supplements, such as vitamin D, selenium, and lycopene, may offer protective benefits against prostate cancer. For instance, lycopene, an antioxidant found in tomatoes, has been shown in several observational studies to reduce prostate cancer risk, though results from randomized controlled trials (RCTs) have been inconsistent. Similarly, selenium supplementation has shown promise in some preclinical studies, yet large-scale clinical trials like the SELECT trial did not demonstrate a clear benefit, and in some cases, selenium supplementation was linked to an increased risk of high-grade prostate cancer. The 5-alpha-reductase inhibitors (5-ARIs), such as finasteride and dutasteride, have shown a reduced incidence of prostate cancer in clinical trials, but concerns about their potential to increase the risk of aggressive forms of the disease persist. These conflicting findings highlight the complexity of prostate cancer prevention, suggesting that while some supplements and pharmacological agents may have potential, further research is required to determine the most effective and safe strategies. Personalized prevention approaches, accounting for genetic and environmental factors, could ultimately offer more reliable solutions for prostate cancer risk reduction^[46,47]^.

### Clinical guidelines and recommendations for prostate cancer prevention

Prostate cancer (PCa) is one of the most common cancers affecting men globally, and its incidence continues to rise, underscoring the need for effective prevention strategies. While prostate cancer screening and early detection have been extensively studied, clinical guidelines regarding prevention remain complex and vary across different health organizations. This narrative reviews the current clinical guidelines and recommendations for prostate cancer prevention, highlighting key strategies that healthcare providers can adopt to reduce the risk of developing prostate cancer.

1. **Lifestyle Modifications**

Lifestyle factors, particularly diet, physical activity, and maintaining a healthy weight, play an essential role in reducing the risk of prostate cancer. Clinical guidelines emphasize the importance of incorporating healthy habits into daily life as part of an overall strategy for cancer prevention.

- **Diet and Nutrition**: The American Cancer Society (ACS) recommends a diet rich in fruits, vegetables, and whole grains, which provide antioxidants and nutrients that may reduce cancer risk. Specific dietary factors such as lycopene, found in tomatoes, and cruciferous vegetables like broccoli and cabbage have shown promising results in lowering prostate cancer risk. However, further evidence is needed before definitive dietary recommendations can be made.
  - **Limiting Red Meat and Dairy**: High consumption of red meat, particularly processed meats, and dairy products has been associated with an increased risk of prostate cancer in some studies. The ACS recommends reducing the intake of these foods and replacing them with healthier protein sources, such as fish, legumes, and plant-based options.
  - **Omega-3 Fatty Acids**: The American Urological Association (AUA) notes that omega-3 fatty acids, particularly from fish oils, may have anti-inflammatory properties that could potentially reduce prostate cancer risk. Incorporating fish, such as salmon and mackerel, into the diet or considering supplementation may offer protective benefits.
- **Physical Activity**: Regular physical exercise is universally recommended as part of prostate cancer prevention. The ACS highlights that men who engage in physical activity, especially aerobic exercises, may lower their risk of developing prostate cancer and have improved survival outcomes if diagnosed with the disease. Exercise helps regulate hormone levels, including testosterone, and decreases chronic inflammation, both of which are implicated in prostate cancer development.
- **Weight Management**: Maintaining a healthy body weight is crucial in preventing prostate cancer. Obesity is associated with a higher risk of aggressive forms of prostate cancer. The AUA recommends that overweight men work towards weight reduction through a combination of diet and physical activity.

1. **Pharmacological Interventions**

Several pharmacological interventions have been explored for their potential to reduce prostate cancer risk, particularly in high-risk individuals. However, while some treatments have shown promise, their use requires careful consideration of the potential benefits and risks.

- **5-Alpha Reductase Inhibitors (5-ARIs)**: Medications such as finasteride and dutasteride, which inhibit the enzyme responsible for converting testosterone to its more potent form, dihydrotestosterone (DHT), have been shown to reduce prostate cancer risk in some studies. The **Prostate Cancer Prevention Trial (PCPT)** demonstrated that finasteride reduced the risk of prostate cancer by about 25%. However, concerns about the possible increased risk of high-grade tumors and sexual side effects have tempered the enthusiasm for widespread use. As a result, the U.S. Preventive Services Task Force (USPSTF) advises that 5-ARIs should be considered only for men at high risk, with a discussion of the potential harms and benefits.
- **Nonsteroidal Anti-Inflammatory Drugs (NSAIDs)**: Chronic inflammation has been implicated in prostate cancer development, and NSAIDs like aspirin have been considered as potential chemopreventive agents. However, clinical evidence on the effectiveness of NSAIDs in preventing prostate cancer is mixed. The ACS suggests that while NSAIDs may offer some protection, the potential side effects, such as gastrointestinal bleeding, limit their routine use for prostate cancer prevention.
- **Vitamin D**: There is accumulating evidence that vitamin D may have a role in preventing prostate cancer, particularly due to its effects on cell differentiation and apoptosis. The American Urological Association notes that men with higher levels of vitamin D have a lower risk of aggressive prostate cancer. Supplementation is recommended for men who have insufficient levels of vitamin D, although routine use for cancer prevention is still under investigation.

1. **Screening and Early Detection**

Screening for prostate cancer remains controversial, as the potential harms of overdiagnosis and overtreatment must be balanced against the benefits of early detection. Current guidelines for prostate cancer screening largely recommend a risk-based approach, considering individual factors such as age, family history, and genetic predisposition.

- **Prostate-Specific Antigen (PSA) Testing**: The USPSTF recommends that men aged 55 to 69 engage in shared decision-making with their healthcare provider regarding PSA testing. This approach emphasizes that the decision to undergo screening should be individualized, weighing the potential benefits and harms, such as false positives, unnecessary biopsies, and treatment-related side effects. PSA testing is generally not recommended for men aged 70 or older or those with a life expectancy of less than 10 years.
- **Digital Rectal Examination (DRE)**: The role of DRE in routine prostate cancer screening is less clear. While it is sometimes performed alongside PSA testing, the AUA suggests that DRE should not be used as a sole screening tool due to its limited sensitivity and specificity.
- **Multiparametric MRI**: For men with elevated PSA levels or abnormal findings on DRE, the use of multiparametric MRI has become an increasingly important tool for determining the need for biopsy. This advanced imaging technique provides greater accuracy in identifying clinically significant prostate cancers and reduces the likelihood of unnecessary biopsies.
- **Genetic Testing**: As understanding of genetic predisposition to prostate cancer improves, clinical guidelines are beginning to incorporate genetic screening, particularly for men with a strong family history of the disease or those who carry mutations in high-risk genes like **BRCA1, BRCA2**, and **HOXB13**. Genetic testing can guide more personalized recommendations for early screening and prevention.

1. **Genetic and Family History Considerations**

Prostate cancer often runs in families, with men who have a first-degree relative with prostate cancer being at increased risk. The National Comprehensive Cancer Network (NCCN) recommends that men with a family history of prostate cancer consider earlier and more frequent screening, beginning at age 40 to 45, depending on the family history. For men with known inherited mutations, such as **BRCA2**, or those with a history of other cancers in the family, targeted screening strategies are critical. Genetic counseling and testing are recommended for individuals with a family history of aggressive prostate cancer, particularly in younger men, as early detection can improve outcomes.

Table 1 shows summary of the key points of the review article on **Prostate Cancer Risk Reduction: Promising Prevention Practices and Insights (provided by the author).**

**Table 1:** **Summary of the key points of the review article on** Prostate Cancer Risk Reduction: Promising Prevention Practices and Insights

Prevention Strategy	Key Findings	Clinical Recommendations
Lifestyle modifications	–Diet rich in fruits, vegetables, whole grains, and omega-3 fatty acids may reduce risk.	–Encourage a healthy, balanced diet with fruits, vegetables, and lean proteins.
	–Reducing red meat and dairy intake linked to lower risk of prostate cancer.	–Advise men to limit red meat and processed dairy products
	–Regular physical activity reduces prostate cancer risk and improves survival outcomes.	–Recommend regular physical exercise, especially aerobic activity
	–Maintaining a healthy weight lowers risk, particularly for aggressive prostate cancer.	–Promote weight management through diet and exercise
Pharmacological interventions	− 5-alpha reductase inhibitors (finasteride, dutasteride) can reduce risk but may have side effects.	–Consider 5-ARIs for high-risk individuals with shared decision-making regarding benefits and harms
	–NSAIDs have mixed evidence for cancer prevention but are not recommended routinely.	–Avoid routine NSAID use for prostate cancer prevention due to potential side effects
	–Vitamin D may offer protective effects but evidence is inconclusive.	–Recommend vitamin D supplementation for individuals with low levels
Screening and early detection	–PSA testing should be based on shared decision-making, particularly for men aged 55-69.	–Offer PSA screening for men aged 55-69 with a personalized discussion of benefits and risks
	–Digital Rectal Examination (DRE) is not recommended alone due to low sensitivity.	–Use DRE only alongside PSA testing, not as a standalone screening tool
	–Multiparametric MRI improves biopsy accuracy in men with elevated PSA.	–Incorporate multiparametric MRI for men with abnormal PSA or DRE findings
Genetic and family history	–Family history and inherited mutations (BRCA2, HOXB13) increase risk, necessitating early screening.	–Consider genetic testing and earlier screening for men with a family history or known genetic mutations
	–High-risk individuals should begin screening at age 40-45.	–Initiate prostate cancer screening early in men with strong family histories or genetic predispositions

## Conclusion

Prostate cancer prevention relies on a multifaceted approach, incorporating lifestyle modifications, pharmacological interventions, risk-based screening, and genetic considerations. Current evidence supports the importance of a healthy diet, regular physical activity, and weight management in reducing prostate cancer risk. Pharmacological agents like 5-alpha reductase inhibitors show promise, but their use requires careful consideration of potential harms. Screening guidelines emphasize shared decision-making, particularly for men aged 55 to 69, with personalized approaches based on family history and genetic risk.

## Data Availability

Not applicable.
